# A positive feedback cell signaling nucleation model of astrocyte dynamics

**DOI:** 10.3389/fneng.2013.00004

**Published:** 2013-07-10

**Authors:** Christopher L. MacDonald, Gabriel A. Silva

**Affiliations:** ^1^Department of Bioengineering, University of CaliforniaSan Diego, La Jolla, CA, USA; ^2^Department of Ophthalmology, University of CaliforniaSan Diego, La Jolla, CA, USA; ^3^Neurosciences Program, University of CaliforniaSan Diego, La Jolla, CA, USA

**Keywords:** calcium, diffusion, astrocytes, modeling, feedback, signaling

## Abstract

We constructed a model of calcium signaling in astrocyte neural glial cells that incorporates a positive feedback nucleation mechanism, whereby small microdomain increases in local calcium can stochastically produce global cellular and intercellular network scale dynamics. The model is able to simultaneously capture dynamic spatial and temporal heterogeneities associated with intracellular calcium transients in individual cells and intercellular calcium waves (ICW) in spatially realistic networks of astrocytes, i.e., networks where the positions of cells were taken from real *in vitro* experimental data of spontaneously forming sparse networks, as opposed to artificially constructed grid networks or other non-realistic geometries. This is the first work we are aware of where an intracellular model of calcium signaling that reproduces intracellular dynamics inherently accounts for intercellular network dynamics. These results suggest that a nucleation type mechanism should be further investigated experimentally in order to test its contribution to calcium signaling in astrocytes and in other cells more broadly. It may also be of interest in engineered neuromimetic network systems that attempt to emulate biological signaling and information processing properties in synthetic hardwired neuromorphometric circuits or coded algorithms.

## 1. Introduction

Calcium signaling in astrocytes participates in a number of biological and physiological functions, including the control of vascular dilation, synapse formation, neurogenesis, and synaptic plasticity. For recent reviews see Araque (2008) and Halassa and Haydon (2010). In addition to intracellular calcium transients, astrocytes can display long range coherent intercellular calcium waves (ICW) engaging upwards of hundreds of cells. These waves are characterized by the generation of intracellular calcium transients in individual cells and calcium induced non-calcium paracrine signaling between cells and across the network. ICW were first observed *in vitro* by Cornell-Bell et al. (1990) following bath application of glutamate to a culture of astrocytes (Cornell-Bell et al., 1990). Under normal physiological conditions, ICW have now been observed *in vivo* in Bergmann glia in the cerebellum radiating from discrete origination sites (Hoogland et al., 2009) and from Muller cells in the neural sensory retina (Kurth-Nelson et al., 2009). ICW have not been observed in cortex under physiological conditions, but have been observed occurring spontaneously under pathophysiological conditions *in vivo* in an APP/PS1 mouse model of Alzheimer's disease (Kuchibhotla et al., 2009). Our group has shown that amyloid-β (Aβ) produces an increase in the frequency of intracellular calcium transients in isolated astrocytes and in the number of cells that display such responses. We also showed that amyloid-β is sufficient to induce ICW in isolated astrocyte networks (Chow et al., 2010). The functional and clinical consequences of such astrocyte network signaling, if any, are not yet known.

Why ICW in astrocytes occur is still poorly understood, nor is it completely clear what are the conditions responsible for when they occur. This is in part due to our lack of an appropriate physiological or pathophysiological context, in the sense that it is difficult to predict when these events will occur if we do not know why they are occurring. The intracellular mechanisms and biochemical pathways that produce such events, however, are relatively well understood. This makes the need for data driven testable models even more important in order to guide experimental thinking and hypothesis formation. Here we wanted to test if a well known set of biochemical pathways when coupled with a simple stochastic rule governing the spatial clustering of calcium release could account for both intracellular calcium transient spikes and ICW. We show that the model is able to simultaneously and accurately reproduce both intracellular and intercellular calcium dynamics. This is an inherent property of the model that requires no modification or tailoring of parameters to one condition or another. Accurate intracellular signaling and intercellular network dynamics emerge naturally from the same mechanisms. These results suggest that a nucleation type process is a very plausible physiological and potentially pathophysiological mechanism that warrants further experimental study. It may also be of interest in engineered neuromimetic network systems that attempt to emulate biological signaling and information processing properties in synthetic hardwired neuromorphometric circuits or coded algorithms.

## 2. Results

### 2.1. Description of the model

**Figure 1:**
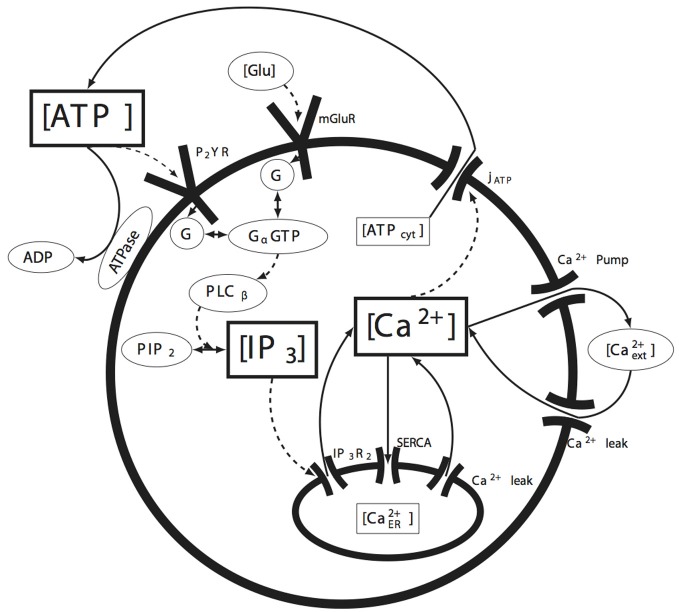
**Summary of the molecular signaling pathways modeled in individual astrocytes**. The solid arrows represent the movement of the indicated molecule (e.g., calcium or ATP), while the dashed arrows represent the action of a species on some downstream effector (e.g., the action or effect of IP_3_ on its receptor IP_3_*R*). The species bounded in the rectangular boxes indicate the state variables tracked by the model. See the text for details.

A schematic of the cell signaling pathways we considered is shown in Figure 1. When either of the P2YR or mGlurR G-protein coupled receptors (GPCRs) are activated through ATP or glutamate at the cell membrane, G-proteins are cleaved producing free *G*_α_ subunits. This in turn activates PLC_β_ which subsequently facilitates the cleavage of phosphatidylinositol 4,5-bisphosphate (PIP_2_) into diacyl glycerol (DAG) and IP_3_. Because IP_3_ receptors are facilitated by calcium in addition to IP_3_, as the concentration of IP_3_ increases, the IP_3_*R* on the endoplasmic reticulum (ER) open, releasing calcium into the cytosol and leading to calcium induced calcium release. As we discuss below, it is a stochastic form of IP_3_*R* clustering in Equation (2) that is responsible for the positive feedback nucleation of calcium micro-events. As calcium increases, a calcium dependent ATP release mechanism releases ATP back into the cytosol, which in some cases results in the intercellular propagation of calcium waves. Other mechanisms modeled include calcium re-sequestration into the ER through the action of a sarcoplasmic-endoplasmic reticulum calcium-ATPase (SERCA) pump, a plasmalemmal calcium-ATPase pump on the cell membrane, ATP degradation in the media due to ATPases, and calcium leak currents through the cell and ER membranes. Gap-junctional connectivity is not modeled. Nor was calcium buffering explicitly taken into account. We discuss this further below. These pathways represent the simplest possible set of biochemical pathways that in our hands were able to produce ICW and reproduce the experimental data. Given the immense complexity of possible intracellular signaling pathways, this model is relatively simple and straightforward, yet is sufficient to account for the data. Parameter values were taken from relevant work in the peer reviewed literature or fits to our own experimental data. Values, data fitting, and sources are listed in Table 1.

**Table 1:** **List of model parameters and values**.

**Parameter**	**Value**	**Units**	**Description**	**References**
**CALCIUM SUBSYSTEM**				
β	0.0244	−	Buffer Ratio	Bennett et al., 2005
*k*_*l*_ext__	1.0e–4	μMs^−1^	Rate of flux due to extracellular [*Ca*^2+^]	fit
*C*_ext_	2000	μM	Extracellular calcium concentration	Valant et al., 1992
*j*_IP3R_max__	100	μMs^−1^	Max rate of IP3R	Fink et al., 1999
*k*_*C*_	0.06	μM	Calcium dissociation constant from IP3R	Fink et al., 1999
*k*_*I*_	0.02	μM	IP_3_ dissociation constant from IP3R	Fink et al., 1999
*k*_off_	0.75	μMs^−1^	Inactivation rate of IP3R due to calcium	Fink et al., 1999
*k*_on_	0.1	μM	Reactivation rate of IP3R	Fink et al., 1999
*j*_SERCA_max__	5.8	μMs^−1^	Maximum rate of SERCA pump activity	Fink et al., 1999
*k*_SERCA_	0.25	μM	Activation [*Ca*^2+^] of SERCA pump	Fink et al., 1999
*R*_vol_	5.4	−	Ratio of volumes of Cytosol:ER	Lemon et al., 2003
*k*_*l*_ER__	6e–4	μMs^−1^	ER leak constant	fit
*k*_1_	0.08	μM	threshold for low activity mode of PCA pump	Valant et al., 1992
*k*_2_	0.38	μM	threshold for high activity of PCA pump	Valant et al., 1992
*V*_1_	1.63	μMs^−1^	maximal PCA pump rate at low activity	Valant et al., 1992
*V*_2_	31.67	μMs^−1^	maximal PCA pump rate at high activity	Valant et al., 1992
**IP_3_ SUBSYSTEM**				
*k*_deg_	0.8	s^−1^	Degradation rate of IP_3_→ PIP_2_	Bennett et al., 2005
r^*^_h_	0.02	μM μm^−2^ *s*^−1^	GPCR kinetic parameter	Bennett et al., 2005
*k*_ATP_	15	μM	Dissociation constant for ATP on P2YR	Bennett et al., 2005
*k*_Glu_	5	μM	Dissociation constant for Glutamate on P2YR	Bennett et al., 2008
*k*_*d*_	0.15	s^−1^	Deactivation rate of free G-protein	Bennett et al., 2005
*k*_*a*_	0.017	s^−1^	Activation rate of free G-protein	Bennett et al., 2005
**ATP DIFFUSION AND RELEASE**				
*D*	150	μm^2^ *s*^−1^	Diffusivity of ATP	MacDonald et al., 2008
α	−0.35	s^−1^	ATP degradation rate	MacDonald et al., 2008
*k*_rel_	0.08	μM	[*Ca*^2+^] for 1/2 maximal ATP release	Fit
*C*_min_	0.02	μM	Min. [*Ca*^2+^] for ATP release	Fit
*j*_ATP_max__	1	μm^2^ *s*^−1^	Max ATP release rate	Fit
σ	0.02	−	Std. Dev. noise	fit

**Figure 2:**
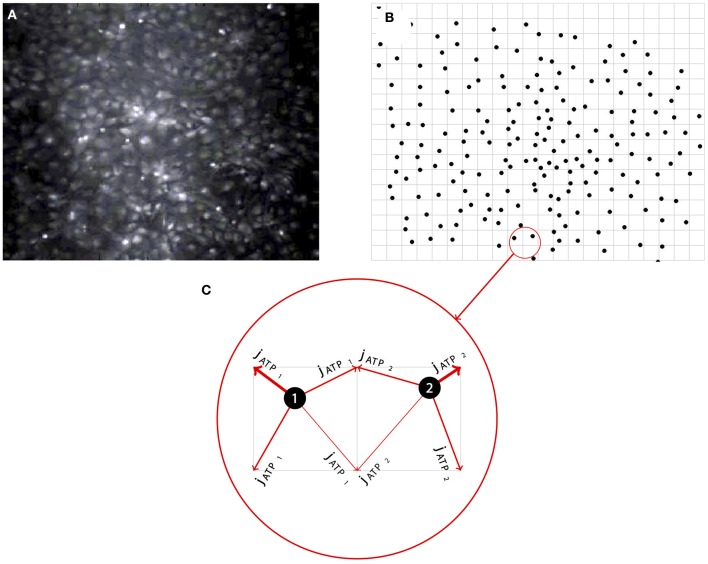
**Representative example of the network simulation approach. (A)** Simulated networks were based on real network geometries derived from the cell locations of imaged fields of spontaneously forming primary dissociated astrocyte networks. **(B)** The locations of each cell as a point source was then mapped onto a regular grid, preserving the native relative spatial relationship between each astrocyte in the visible network. **(C)** To run the simulations, modeled ATP diffusing from each cell (i.e., the intercellular paracrine component of the signaling cascade) was distributed between the four nearest neighbor grid points weighted by the distance to those grid points.

In the model, there are *N* cells in an M-dimensional space at locations ${\vec{x}}_{i}=\left\{x_{1},\;x_{2}\;\ldots \;x_{M}\right\}$, where *i* = {1 … *N*}. Each cell is regarded as a single compartment model that upon excitation releases a point source of ATP at its location which diffuses between and activates other cells in the simulated field (Figure 2; see section 4 below also). There are five state variables: Intracellular calcium *C*_*i*_(*t*), intracellular IP_3_*I*_i_(*t*), extracellular ATP $A(\vec{x},\;t)$, ER calcium *C*_ER__*i*_(*t*), and the gating state variable of the IP_3_*R* *h*_*i*_(*t*). The governing state equations are given by:

(1) $$\frac{dC_{i}}{dt}=\beta (j_{\text{IP3R}}-j_{\text{SERCA}}+j_{l_{\text{ER}}}-j_{\text{PCA}}+j_{l_{\text{ext}}})$$

(2) $$\frac{dh_{i}}{dt}=k_{\text{off}}(k_{\text{on}}-(C_{i}+k_{\text{on}})h)$$

(3) $$\frac{dC_{{\text{ER}}_{i}}}{dt}=R_{\text{vol}}(j_{\text{SERCA}}-j_{\text{IP3R}}-j_{l_{\text{ER}}})$$

(4) $$\frac{dI_{i}}{dt}=r_{h}^{*}\left(G_{\text{ATP}}^{*}+G_{\text{glu}}^{*}\right)-k_{\text{deg}}I_{i}+dt^{\frac{1}{2}}\eta_{i}(\sigma ,\;t)$$

(5) $$\frac{\partial A(\vec{x})}{\partial t}=D\nabla^{2}A-\alpha A+\sum_{i}\delta_{\vec{x},\;{\vec{x}}_{i}}j_{{\text{ATP}}_{i}}$$

#### 2.1.1. Intracellular calcium *C*_*i*_(*t*)

The free calcium concentration in the cytosol is governed by intracellular calcium in the ER entering (*j*_IP3R_ and *j*_*l*_ER__) and leaving (*j*_SERCA_) the cytosol, and by extracellular calcium entering (*j*_*l*_ext__) and leaving (plasmalemmal Ca^2+^ ATPase, *j*_PCA_). β is a term for steady state calcium buffering in the cytosol. This formulation is similar to the work by Bennett et al. (2005) and is assumed to be constant. The leak from the extracellular space is given by

(6) $$j_{l_{\text{ext}}}=k_{l\text{ext}}\left(1-\frac{C_{i}}{C_{\text{ext}}}\right)$$

where *C*_ext_ is the calcium concentration in the medium, *k*_*l*ext_ is the maximum leak rate, and the *Ca*^2+^−ATPase pump dynamics is described as

(7) $$j_{\text{PCA}}=V_{1}\frac{C_{i}^{1.7}}{k_{1}^{1.7}+C_{i}^{1.7}}+V_{2}\frac{C_{i}^{4.4}}{k_{2}^{4.4}+C_{i}^{4.4}}$$

as developed in Valant et al. (1992) and represents different activity levels in the basal and excited states. The equation for the IP_3_*R* is based on Fink et al. (1999).

(8) $$j_{\text{IP3R}}=j_{{\text{IP3R}}_{\text{max}}}{\left(\frac{C_{i}}{C_{i}+k_{C}}\frac{I_{i}}{I_{i}+k_{I}}h_{i}(t)\right)}^{3}\left(1-\frac{C_{i}}{C_{{\text{ER}}_{i}}}\right)$$

where *j*_IP3R__max_ is the maximum rate of calcium diffusion from the ER, *k*_*C*_ and *k*_*I*_ are the activation concentrations of cytosolic calcium and IP_3_, respectively. *h*_*i*_(*t*) is a gating variable which represents calcium based inactivation of the receptor. The calcium is pumped back into the ER through the SERCA pump at a rate given by

(9) $$j_{\text{SERCA}}=j_{{\text{SERCA}}_{\text{max}}}\frac{C_{i}^{2}}{C_{i}^{2}+k_{\text{SERCA}}^{2}}$$

where *j*_SERCA__max_ is the maximum pump rate and *k*_SERCA_ is the activation concentration of calcium for the pump (Adapted from Fink et al., 1999). There is also a leak current through the ER given by

(10) $$j_{l_{\text{ER}}}=k_{l_{\text{ER}}}\left(1-\frac{C_{i}}{C_{{\text{ER}}_{i}}}\right)$$

where *k*_*l*_ER__ is the rate of calcium leak through the ER.

It is also necessary to track the store of calcium in the ER. In Equation (3) *R*_vol_ is the ratio of the volume of the cytosol to the volume of the ER, with the other terms as described above.

#### 2.1.2. Intracellular IP_3_ concentration *I*_*i*_(*t*)

The governing equation for IP_3_ concentration has two terms, one for generation (PIP_2_ ⇒ IP_3_) due to the G-protein signaling cascade and one for degradation (IP_3_ ⇒ PIP_2_) at a rate *k*_deg_. The parameter r^*^_h_ is a part of the G-coupled protein kinetics, and G^*^ is the steady state level of free G-protein in the cell. The steady state level of G-protein is given by the sum of G-protein created through external ATP and glutamate where

(11) $$G_{\text{ATP}}^{*}=\frac{\rho_{\text{ATP}}+\delta }{\frac{k_{d}}{k_{a}}+\delta +\rho_{\text{ATP}}}$$

(12) $$\rho_{\text{ATP}}(i,\;t)=\frac{A({\vec{x}}_{i},\;t)}{k_{\text{ATP}}+A({\vec{x}}_{i},\;t)}$$

(13) $$\delta =\frac{\frac{k_{d}}{k_{a}}k_{\text{deg}}I_{0}}{r_{h}^{*}-k_{\text{deg}}I_{0}}$$

describe the free G-protein due to enzymatic activity of activated PLC_β_, downstream of activation of metabotropic P2YRs by external ATP, and is adapted from the work of Bennett et al. (2005). $A(\vec{x},\;t)$ is the concentration of ATP at the location of cell *i*, *k*_ATP_ is the dissociation constant of ATP binding to the P2YRs, *k*_*a*_, and *k*_*d*_ are the activation and deactivation rates of G-protein, δ is the ratio of the activities of the bound and unbound receptors, and *I*_0_ is the steady state initial concentration of IP_3_ in the cell. The equation describing free G-protein due to mGluRs is given by

(14) $$G_{\text{glu}}^{*}=\frac{\rho_{\text{glu}}}{\frac{k_{d}}{k_{a}}+\rho_{\text{glu}}}$$

(15) $$\rho_{\text{glu}}=\frac{{\left({\left[\text{glu}\right]}_{\text{ext}}\right)}^{0.7}}{{\left(k_{\text{glu}}\right)}^{0.7}+{\left({\left[\text{glu}\right]}_{\text{ext}}\right)}^{0.7}}$$

The formula for ρ_glu_ was adapted from Bennett et al. (2008) with the added modification of a Hill coefficient of 0.7 as derived in De PittÃ et al. (2009) modeling negative cooperativity of glutamate binding to multiple mGluR subunits.

Previous work by Shuai and Jung (2002) derived a stochastic form of the Li-Rinzel model for small IP_3_*R* clusters to describe calcium puffs and showed it mirrored a first principles Markov-based approach. This was accomplished by adding a noise term to the gating variable for small clusters of IP_3_Rs on the ER. In this whole cell model, small clusters of activating IP_3_Rs would result in noise in the whole cell IP_3_ signal, and is represented here by a white noise term in the IP_3_ equation. η _*i*_(*t*) is a zero-mean Gaussian white noise term with standard deviation σ. The $dt^{\frac{1}{2}}$ term ensures that the effect of the noise term on the system will remain constant regardless of the time step in the simulation.

#### 2.1.3. Extracellular ATP concentration $A(\vec{x},\;t)$

The concentration of ATP in the media can change through several mechanisms. At the location of a cell, we model the ATP release (*j*_ATP_). ATP is also degraded in the media at a rate α, and diffuses through the media with a diffusivity *D* (*μ*m^2^s^−1^). δ is the Kronecker delta function such that $\delta_{\vec{x},\;{\vec{x}}_{i}}=1$ when $\;\vec{x}={\vec{x}}_{i}$ and is zero otherwise, so that *j*_ATP__*i*_ is only added at the location of cell *i*. For ATP release we will use a general Hill model of calcium dependent release as in Bennett et al. (2005).

(16) $$j_{{\text{ATP}}_{i}}=\{\begin{array}{cc} j_{{\text{ATP}}_{\text{max}}}\frac{C_{i}-C_{\text{min}}}{k_{\text{rel}}+C_{i}}, & C_{i}>C_{\text{min}}\\ 0, & C_{i}<C_{\text{min}} \end{array}$$

*C*_min_ is the minimum threshold for ATP current, and *k*_rel_ is the hill coefficient.

#### 2.1.4. Positive feedback nucleation is mediated by the IP_3_*R* gating variable, *h*_*i*_(*t*)

Calcium nucleation events occur in the model because we used a stochastic form of the h-gate equation (Equation 2) for describing the kinetics of the calcium channel opening and closing on the ER membrane. Generally, for dynamics of homogeneous whole cell models, one assumes the cell behaves like a continuously stirred reactor, since due to the large number of receptors and ions involved the cell can be represented by deterministic dynamical variables. Whole cell models of calcium dynamics generally follow this trend (Bennett et al., 2005; Lavrentovich and Hemkin, 2008). More detailed and complex internal dynamics are left to intracellular models which track the diffusion and release of different species within the cell. However, this assumption of homogeneity is not valid for models of the IP_3_*R* for many cell types including astrocytes, since experimental observations show that global calcium elevations are a stochastic phenomenon that arise from individual localized events within the cell (Perc et al., 2008; Skupin et al., 2008). Intracellular modeling studies such as the work by Skupin et al. (2008) suggest that this is due to the local nucleation of calcium waves resulting from the stochastic opening of small clusters of IP_3_ receptors. Their work showed that on the scale of a small cluster of about ten to forty IP_3_*R*, the fluctuations of gate opening and closing can lead to a random local spike in calcium which can then propagate throughout the cell, creating a global calcium elevation. In Equation (2) *k*_off_ is the inactivation rate due to calcium binding to the inhibitory site and *k*_on_ is the corresponding reactivation rate.

### 2.2. Aperiodic intracellular calcium transient oscillations in individual astrocytes

**Figure 3:**
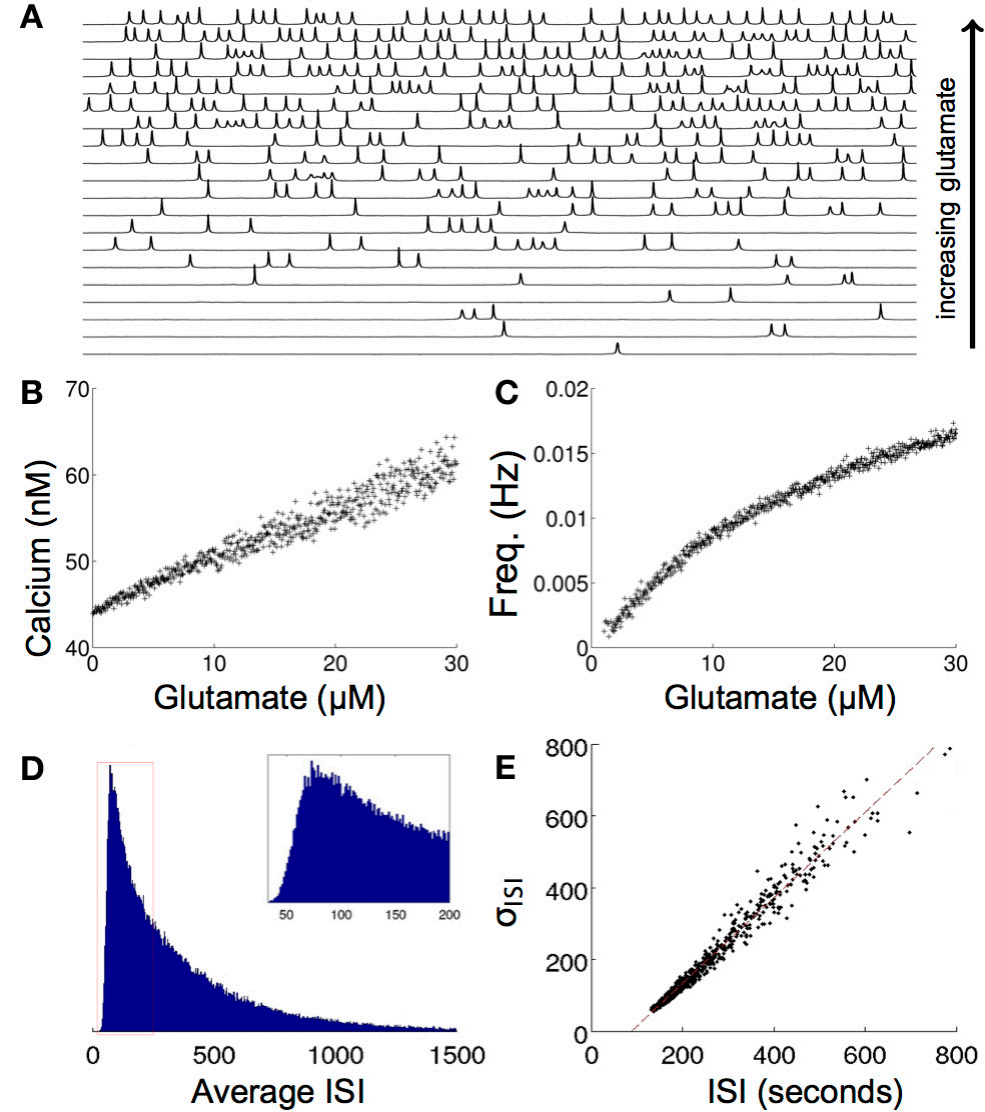
**Effects of increasing glutamate concentration on a single modeled astrocyte. (A)** Increasing glutamate induces spontaneous oscillations in a single cell. **(B,C)** As the level of applied glutamate increases, both the baseline calcium between excitation and the frequency of excitation increase. **(D)** A histogram of the ISI of one run for 5x10^7^ s shows a poisson distribution of the ISI, as is predicted from stochastic intracellular modeling. **(E)** The σ_ISI_−ISI_av_ plot of many runs of different glutamate concentrations (0–50 μM) shows a linear relationship as seen experimentally.

Modeling the effects of exogenous glutamate in single cell simulations resulted in intracellular calcium transients with an oscillatory regime with a random period every 50–600 ms as a function of the concentration of glutamate. Some astrocytes have been observed to undergo calcium transient oscillations *in vivo*. Periodic oscillations have been seen in thalamic astrocytes (Parri and Crunelli, 2001), although most astrocytes display random aperiodic oscillations (Cornell-Bell et al., 1990; Skupin et al., 2008). The mechanisms that produce these dynamics are not completely clear. Figure 3A shows a raster plot of the calcium transient responses for a number of atrocytes in the network organized from high to low firing frequencies. There is a qualitative aperiodic variation in the firing patterns for different cells in the network in response to these conditions. This is consistent with experimental data (Skupin et al., 2008). Changing the concentration of glutamate has a relatively linear effect on the resultant concentration of calcium release. As the glutamate level rises, the baseline concentration of calcium follows it (Figure 3B). This in turn produces an increase in the firing frequency itself (Figure 3C). The model was run with σ = 0.02, where σ is standard deviation from baseline noise. The value σ was chosen so that the random poisson distribution of calcium signaling spike events matched the measured distribution of experimental astrocyte recordings (Skupin et al., 2008). Figure 3D shows one representative distribution of the average interspike interval (ISI) for one particular run over a period of about 1500 s. Of particular note, experimental measurements of intracellular oscillations have shown that the slope of the curve of the standard deviation of interspike interval (σ_ISI_) vs. average interspike interval (*T*_av_) is linear over many parameters for different cell types, including astrocytes (Skupin et al., 2008; Skupin and Falcke, 2009), an effect that was reproduced by our model (Figure 3E). Intracellular biophysical models (Skupin and Falcke, 2009) but not deterministic point source models (Lavrentovich and Hemkin, 2008) predict this. Essentially, the probability of the initiation of a local nucleation is a poisson distribution, which after an initial refractory period following an event is time-independent. This type of probability distribution of spiking results in a linear σ_ISI_ vs. *T*_av_ curve.

### 2.3. Temporally delayed spontaneous intercellular calcium waves

**Figure 4:**
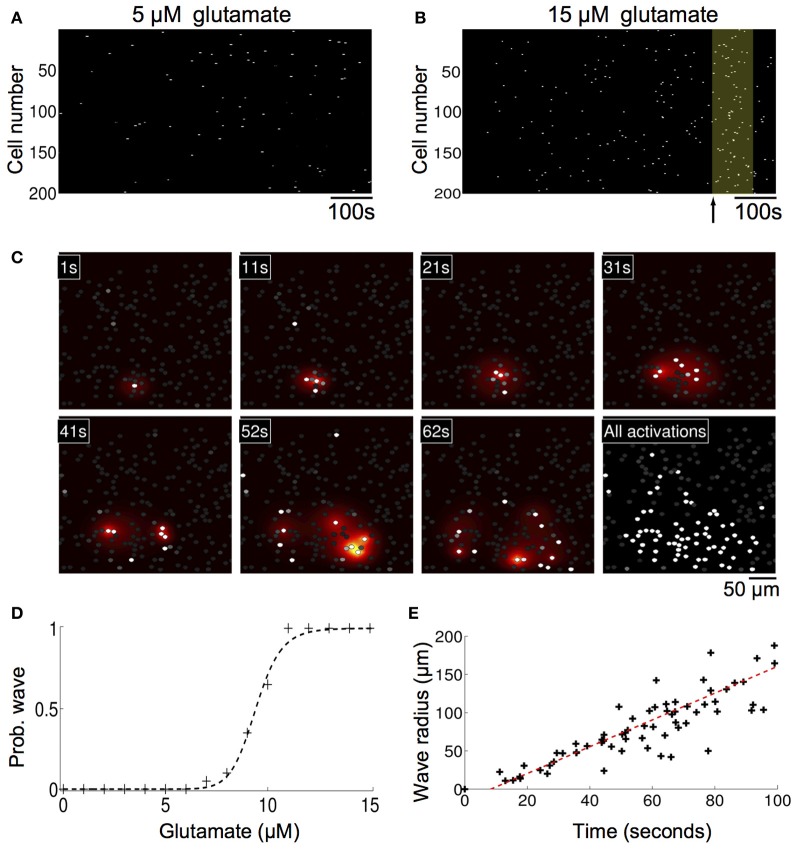
**Astrocyte network simulations of the signaling nucleation model exposed to a glutamate stimulus resulted in temporally delayed spontaneous intercellular calcium waves (ICW). (A)** Rater plot of cell activations as a function of time for astrocytes in the network. Stimulation with 5 μM glutamate produced spontaneous transients in individual cells but no coherent wave like activity. **(B)** At a glutamate concentration of 10 μM, however, temporally delayed ICW appeared in the network, i.e., relative to the time of glutamate stimulation at time zero. In the example shown here a wave was observed (yellow box) beginning 620 s (arrow) after the stimulation. **(C)** Spatial detail of the wave shown in panel (**B**). Each panel indicates the time point of the progression of the wave. Individual astrocytes participating in the ICW are shown as white dots, while the heat color map shows the spatial diffusion of extracellular ATP (increasing concentrations indicated by the progression from yellow to red). The last panel shows all the astrocytes that participated in the wave. **(D)** The probability of observing an ICW over a 1000 s period as a function of glutamate concentration. There is a sharp transition boundary between about 7–11 μM. **(E)** Plot of the expanding wave front radius as a function of time for the wave shown in panels **(B,C)**. Each black cross indicates the time at which a participating astrocyte activated. There is a strong linear relationship between the wave front and the time it takes to expand.

Without any modifications to the equations or parameters, the model inherently generated spontaneously forming intercellular ICW in simulated astrocyte networks that accurately reproduced the initiation, propagation, and termination of measured experimental waves. Glutamate concentrations below 10 μM resulted in an increase in the number of cells that displayed uncorrelated individual intracellular transients and oscillations, which is expected given the results above, but no coherent ICW (Figure 4A). However, at 10 μM glutamate, temporally delayed coherent ICW nucleated and traveled through the network, analogous to experimental results showing a similar phase transition type event where coherent ICW propagate following an observed period of incoherent and uncorrelated calcium transients in individual astrocytes in a network (Figure 4B) (Cornell-Bell et al., 1990; Kim et al., 1994; MacDonald et al., 2008; Chow et al., 2010). In the same way observed in cell experiments, in the simulations initially calcium oscillations in individual cells were random and uncorrelated. However, later in the simulation at 620 s, independent of any external inputs or perturbations, an ICW emerged (Figure 4B). Figure 4C shows the spread of the ICW wave. Increases in intracellular calcium in individual participating cells (white) and the diffusion of the associated secreted ATP (heat map color coded) illustrate the radial propagation of the wave from a point source. Qualitatively, these results agree with the expected phenomenology of the model given its construction; waves resulted due to localized elevations of extracellular ATP from randomly excited cells, which increased the probability of nearby astrocytes becoming activated and releasing ATP. This in turn led to a positive feedback phenomenon of more and more cells in an area becoming active, eventually supporting an intercellular wave. Below this threshold there were not enough temporally and spatially localized events to elevate the local ATP concentration. In fact, the relationship between the simulated concentration of glutamate and the probability of observing a wave in a 1000 ms time period follows a relatively steep sigmoidal phase transition. Between roughly 7–11 μM the probability of a wave being spontaneously initiated goes from zero to almost certainty very quickly (Figure 4D). In the representative example shown in Figure 4 the waves spread almost 200 μm through the network before ending on their own. This is an important consideration and is in contrast to other modeling attempts where wave like events continue indefinitely unless forced to terminate or until they reach the physical edge of the network. A plot of distance versus time for this particular simulation is shown in Figure 4E. There is a well behaved linear relationship between the size of the expanding wavefront and the time it takes to propagate.

**Figure 5:**
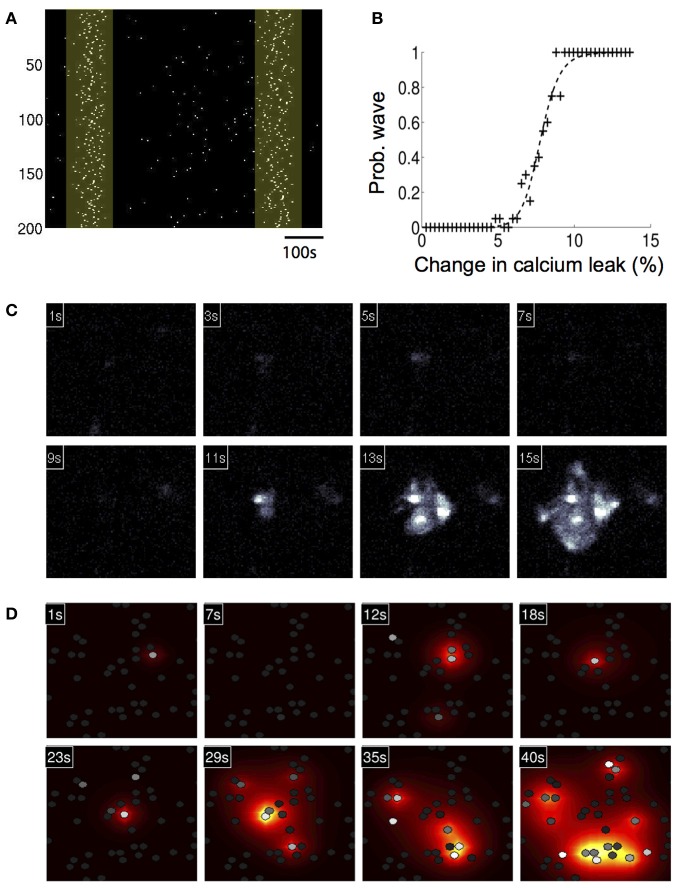
**Astrocyte network simulations of the signaling nucleation model exposed to an increased calcium leak currents that physically models the exposure to amyloid beta peptides also resulted in temporally delayed spontaneous intercellular calcium waves (ICW). (A)** A calcium activation raster plot for participating astrocytes in a network for one simulation following increase to the calcium leak current. Two ICW were observed to occur at just about 100 and 650 s (highlighted boxes). **(B)** Plot of the probability of calcium leak induced ICW waves in a 1000 s period. Beyond about an 8% of a change in the leak current ICW are essentially guaranteed. **(C)** A representative wave showing the spread of intracellular calcium in response to the ICW at different times during the course of the simulation. Each panel indicates the time point of the progression of the wave. **(D)** Intercellular spread of ATP for the same wave from panel **(C)**. The heat color map shows the spatial diffusion of extracellular ATP (increasing concentrations indicated by the progression from yellow to red).

We then investigated whether a simulated increase in extracellular calcium permeability could reproduce experimentally observed ICW induced by exposure to amyloid-beta (Aβ), a peptide who's accumulation in the brain is associated with Alzheimer's disease. Our group has shown that the 1–40 (Aβ) protein fragment is sufficient to induce long range temporally delayed spatially complex ICW in purified cortical astrocyte cultures (Chow et al., 2010). Similar astrocyte calcium waves have been observed to occur spontaneously under pathophysiological conditions *in vivo* in an APP/PS1 mouse model of Alzheimer's disease (Kuchibhotla et al., 2009). From a physical chemistry perspective, Aβ has been shown to induce ion-channel like pores in lipid bilayers (Arispe et al., 1993; Green et al., 2004; Mobley et al., 2004; Quist et al., 2005). Several authors (Lin et al., 1999, 2001) have shown that *A*β 1–40 and 1–42 fragments can induce increased intracellular calcium levels through an *A*β induced calcium current across the cell membrane, which is consistent with the formation of ion-channel like structures permeable to calcium. To test this, we represented the *A*β pore as an increased external calcium leak current from the extracellular space. This was done by increasing the leak current (*k*_*l*_ext__) by 20% to 8.4 × 10^−4^. This number was chosen phenomenologically, since it put the model in a regime where oscillations in single cells and waves in networks were both spontaneously observed. Unfortunately, the magnitude of Aβ induced ionic currents has not been measured experimentally that we know of. With the model modified in this way, the basal calcium and thus sensitivity of the system increased. This means that under conditions of simulated elevated Aβ, when an astrocyte oscillates, more ATP is released into the extracellular space, increasing the chances of nearby cells oscillating, resulting in localized nucleation events that produce ICW. In individual modeled astrocytes this process induced aperiodic calcium transient oscillations similar to those produced by the model in response to elevated glutamate, and the observed distribution of spike timings is also Poissonian, with the σ_ISI_ vs. *T*_av_ relationship similarly linear (data not shown). Figure 5A shows a calcium activation raster plot for participating astrocytes in a network for one simulation. Two ICW can be clearly observed to occur at just over 100 and 650 s following increases to the calcium leak current. The probability of observing such waves in a 1000 s period plotted against the increase in calcium leak due to simulated Aβ showed a well behaved increasing sigmoidal relationship, where beyond a given threshold (about 8% of a change in the leak current) ICW are essentially guaranteed (Figure 5B). Figure 5C shows representative panels of the spread of intracellular calcium in response to the ICW at different times during the course of the simulation, while Figure 5D shows the intercellular spread of ATP. Both qualitatively and quantitatively the simulated waves produced by the model have a strong and reproducible similarity to experimentally measured and observed waves. A typical wave in the simulations ranged from a speed of 1.8–5.1 μM/s depending on the level of stimulation, similar to glutamate and Aβ induced waves in culture, which we have previously reported to have speeds of 4.5–8 μM/s (Chow et al., 2010). This is in contrast to mechanically stimulated waves observed in glial networks which tend to be faster at around 20 μM/s (see for example Yu et al., 2009).

## 3. Discussion

In this work we showed the theoretical plausibility of how a local region of elevated extracellular ATP from excited astrocytes in geometrically realistic networks can increase the probability of nearby cells activating, leading to a positive feedback phenomenon which results in an intercellular wave. Specifically, we showed how this can be achieved by glutamate and Aβ at concentrations typically seen *in vivo*. Experimental observations and intracellular modeling studies have shown whole cell calcium elevations nucleate locally through the stochastic opening of small clusters of IP_3_Rs microdomains (Perc et al., 2008; Skupin et al., 2008). During basal levels of activity under physiological conditions in the cortex, small microdomain increases in calcium predominate, occasionally spreading throughout a whole cell and more rarely between neighboring cells (Fiacco et al., 2009). Long distance waves have not been observed *in vivo* in cortical astrocytes under physiological conditions but have been observed in cortex under pathophysiological conditions (Kuchibhotla et al., 2009) and under normal conditions in the cerebellum and retina (Hoogland et al., 2009; Kurth-Nelson et al., 2009). Our work here suggests that although there is a large difference in scale between a microdomain calcium elevation (~50 nm) and an ICW (~100 μM), both intracellular and extracellular events can be produced by the *same* molecular pathways via a positive feedback that leads to the propagation of the signal. From a dynamical systems perspective this is interesting because it suggests that the system is spatially multi-scale and near a critical threshold between different states, which has been suggested as a common property throughout the brain (Wang et al., 2010), and which is a hallmark of complex phenomena that display emergent properties where the outputs or behaviors of the system cannot be readily predicted by an understanding of the dynamics of the constituent components that make it up. It is possible that other cell types through a variety of cell signaling mechanisms may contribute to the boundary (i.e., initial) conditions or temporal development of the internal state of an astrocyte. The integration of paracrine signaling from other cells such as neurons with the astrocyte's own internal biochemistry likely result in complex non-linear summation processes that may stochastically tip the balance near a critical threshold transition between intracellular states that result in nucleation events versus those that do not. Fully exploring this would likely provide significant insights into in particular reciprocal signaling between neurons and astrocytes under both physiological and pathophysiological conditions. This work follows a previous phenomenological model we published on astrocyte network signaling that addressed an apparent discrepancy in the experimental literature (MacDonald et al., 2008).

There are several other calcium signaling mechanisms in astrocytes and related glial cell types beyond the dominant IP_3_ ATP pathway we consider here that may contribute to the cell biology and physiology of calcium signaling in these cells, including possibly to the initiation, propagation, and dynamics of the nucleation mechanisms we describe. For example, astrocytes, oligodendrocytes in the central nervous system, Schwann cells in the peripheral nervous system, and glial progenitor cells can express voltage gated *Ca*^2+^ channels (MacVicar, 1984; Barres et al., 1990; Amédée et al., 1991; Verkhratsky and Kettenmann, 1996; Lalo et al., 2011; Parpura and Verkhratsky, 2012b), while Bergmann glia in the cerebellum and microglia express ligand gated receptors coupled to metabotropic receptors that regulate calcium release (Kirischuk et al., 1995a,b; Verkhratsky and Kettenmann, 1996; Navarrete and Araque, 2008, 2010). Astrocytes and oligodendrocytes are capable of expressing low voltage T-type and high voltage possibly L-type calcium channel currents at least in culture (MacVicar, 1984; Barres et al., 1989; Berger et al., 1992; Steinhäuser et al., 1992). Of particular physiological significance are the potential implications of these mechanisms on neuronal-glial reciprocal signaling. The activation of voltage gated *Ca*^2+^ channels necessitates a depolarization of the cell membrane, which for glial cells can occur as a result of extracellular potassium elevation following localized neuronal activity. Physiological increases up to about 15 mM in the concentration of extracellular potassium from neurons is sufficient to reach the threshold potential of at least low voltage *Ca*^2+^ channels in glial cells (Kirischuk et al., 1995c). One intriguing and persistent open question is whether astrocytes may use such a mechanism to detect and integrate (and possibly modulate?) neuronal activity (Perea and Araque, 2005, 2006; Stevens, 2008; Eroglu and Barres, 2010; Perea and Araque, 2010). Intracellularly, calcium currents in astrocytes may be mediated by cAMP-dependent phosphorylation (MacVicar and Tse, 1988). Ligand gated *Ca*^2+^ channels may be mediated by GABA_*A*_ or glutamate receptors, both of which are expressed by astrocytes and oligodendrocytes and are central to astrocyte calcium signaling and the modulation of neuronal signaling (Parpura et al., 2011; Parpura and Verkhratsky, 2012a,b, 2013; Verkhratsky et al., 2012; Parnis et al., 2013). Some sub-types of astrocytes and Bergmann glia express kinate/AMPA specific glutamate receptors (Borges et al., 1994; Kirischuk et al., 1995a,b; Porter and McCarthy, 1995; Verkhratsky and Kettenmann, 1996). In addition, recent work by Goldberg et. al. has examined the role of non-linear gap junctions in calcium wave propagation (Goldberg et al., 2010). Small changes in IP_3_ do not result in diffusion to neighboring cells, thus preserving the local independence of cells relative to linear gap junctions. There is on-going discussion as to whether the propagation of ICW are due mostly to intercellular ATP or intracellular diffusion of IP_3_ through gap junctions. Different regions of the brain express different levels of gap junctions, and their degree of contribution may vary from region to region. If the system described here were predominately driven by non-linear gap junctional diffusion as proposed in Goldberg et al. (2010), it would be anticipated that some aspects of our results might be different. Nonetheless, the calcium nucleation phenomena we modeled would stay the same since it is driven by the dynamics of calcium induced calcium release from the ER. Test simulations incorporating a number of these alternative astrocyte calcium signaling pathways into our model did not change the results or our interpretation of the results, although it is likely that the kinetics of some of the reactions might be different as the complexity of the model increases. But as we point out in the beginning of the paper, the goal was to determine the *simplist* set of pathways that could naturally reproduce the stochastic dynamics of the measured data, which are faithfully captured by the IP_3_ ATP pathways we take into account. As such, we omitted these other signaling mechanisms from the current model to avoid extra layers of computational complexity that added little to the resultant analysis. Nonetheless, a more complete investigation of these other pathways in the context of the results we present here will almost certainly provide additional insights worth exploring. This may be particularly true and of considerable importance when considering the potential role of astrocyte network signaling on the modulation and integration of signaling and information processing by neuronal networks, for which very little is known or understood (Araque and Navarrete, 2010; de Pittà et al., 2012).

Application of amyloid beta to mixed cultures of glia and neurons results in widespread cell death within 24 h (Abramov et al., 2003, 2004), and an astrocyte specific role in Alzheimer's neurodegeneration pathways was suggested by these authors. This was partially due to depression of glutathione (GSH) production in the astrocytes, which is required for neuronal survival, induced by disrupted calcium homeostasis. In addition, in our hands (Chow et al., 2010) Aβ induces long range ICW in cultured astrocytes as well as robust reactive gliosis. The justification for modeling the effects of *A*β in our model as an increase in the calcium leak current is based on what is known about the physical effects of *A*β on the membranes of cells. Studies from the lab (Lin et al., 1999, 2001; Bhatia et al., 2000) have shown that free *A*β undergoes conformation changes when interacting with lipid membranes which subsequently result in structures that display ion channel-like properties. Increased intracellular calcium was blocked by anti-*A*β antibody and by depleting the extracellular media of calcium, supporting this view. In addition to the observed increases in intracellular calcium due to *A*β exposure, atomic force microscopy (AFM) imaging of cell membranes has shown ion-channel like structures due to the addition of multiple amyloid proteins (Green et al., 2004; Quist et al., 2005) characterized by “donut” shaped features with a central hole and higher edges. Complimenting such studies, theoretical models have shown that *A*β can form multimers in lipid membranes with a central, calcium permeable pore (Mobley et al., 2004).

## 4. Materials and methods

### 4.1. Experimental data

All experimental data in this paper was previously reported by our group in Chow et al. (2010).

### 4.2. Parameters and simulations

Simulated networks were based on real network geometries, with the cell locations taken from experimental *in vitro* fields and put onto a two-dimensional grid as point sources of ATP that sense the ATP surrounding them, similar to our approach in MacDonald et al. (2008) (Figure 2A). ATP released from each cell by the current *d*A/dt was then added to the four grid points surrounding the cell weighted by the distance of the grid point to the cell center (Figures 2B,C). The grid was divided up into a 2 μ grid and an FTCS (forward time centered space) scheme was run to solve the diffusion equations. In some cases simulations were periodically run with a very fine grid and time step and compared to a coarser solution step size in order ensure numerical stability and accuracy. In all cases the solutions for both always turned out equivalent. The state equations were solved using the forward Euler method. Simulations were also run on randomized fields of cells at the same density and minimum distance from each other as the experimental locations. This led the average distance to the nearest cell to be similar to experimental cell groupings. The initial conditions for *I*_0_, *C*_0_, *A*_0_, and *h*_init_ were chosen by running the simulation once with no stimulation to find steady state variables for a given parameter set. In the case of parameter sets in which cells were spontaneously oscillating and there was no steady state, initial conditions were chosen from a quiescent period during the simulation. The initial value for *C*_*ERinit*_ was set to 400 μM. Initially, gap junctional connectivity (*W*) was set to zero. All the Matlab source code for running these simulations is available upon request. Some parameters were chosen from the relevant literature and some were fit. *k*_*l*_ER__ was chosen to balance the steady state flux against the IP3R and SERCA currents when no system noise was present. *C*_min_ was chosen as a value twice the steady state level of calcium in the cell so there was no ATP leak at steady state and *k*_rel_ was chosen as the half maximal calcium value during a transient. This mirrors the choices for IP_3_ dependent release in Bennett et al. (2005). δ was fit such that beginning at a basal level of glutamate or Aβ an induced leak would occasionally oscillate at a rate we determined from control fields of astrocytes in experiments (see Chow et al., 2010). Values, data fitting, and sources are listed in Table 1.

### Conflict of interest statement

The authors declare that the research was conducted in the absence of any commercial or financial relationships that could be construed as a potential conflict of interest.
